# Enhancing 360 Video Streaming through Salient Content in Head-Mounted Displays

**DOI:** 10.3390/s23084016

**Published:** 2023-04-15

**Authors:** Anh Nguyen, Zhisheng Yan

**Affiliations:** Department of Information Sciences and Technology, School of Computing, George Mason University, Fairfax, VA 22030, USA; anguy59@gmu.edu

**Keywords:** 360 video, video streaming, head movement prediction, saliency detection

## Abstract

Predicting where users will look inside head-mounted displays (HMDs) and fetching only the relevant content is an effective approach for streaming bulky 360 videos over bandwidth-constrained networks. Despite previous efforts, anticipating users’ fast and sudden head movements is still difficult because there is a lack of clear understanding of the unique visual attention in 360 videos that dictates the users’ head movement in HMDs. This in turn reduces the effectiveness of streaming systems and degrades the users’ Quality of Experience. To address this issue, we propose to extract salient cues unique in the 360 video content to capture the attentive behavior of HMD users. Empowered by the newly discovered saliency features, we devise a head-movement prediction algorithm to accurately predict users’ head orientations in the near future. A 360 video streaming framework that takes full advantage of the head movement predictor is proposed to enhance the quality of delivered 360 videos. Practical trace-driven results show that the proposed saliency-based 360 video streaming system reduces the stall duration by 65% and the stall count by 46%, while saving 31% more bandwidth than state-of-the-art approaches.

## 1. Introduction

Virtual Reality (VR) technology has developed rapidly in recent years. Studies [1,2] have shown that the market value of VR is expected to reach $44.7 billion by 2024. In addition, the continuous advancement of VR devices such as head-mounted displays (HMDs) and 360-degree cameras has opened the door for an increasing number of VR applications.

A popular VR application is 360 video streaming [3,4]. A 360 video is created from a 360-degree camera capturing the content in all directions. The streaming server re-encodes the video in fixed-length chunks and sends them to the client upon request. Once the client downloads the video, the video is projected in an HMD as a sphere centered at the user’s head. The user freely looks around to explore the video content as if they are actually inside the sphere [5]. This creates a unique and immersive experience differentiating 360 video streaming from regular 2D video streaming.

In spite of its promising nature, current 360 video streaming systems are expensive and inefficient in terms of bandwidth because of the need for transporting ultra-high-resolution data [6,7,8,9]. Popular streaming platforms such as YouTube and Facebook require the entire 360 video frame to be delivered. However, only a part of the frame is actually displayed in the user’s viewport. As a result, a significant amount of precious bandwidth is wasted on downloading unusable data. A natural way to reduce the bandwidth consumption is spatial fetching, in which video regions that are likely to be viewed by users are streamed in high quality while the remaining regions are either ignored or downloaded in low quality. To enable spatial fetching, a head-movement prediction model must be employed to determine where the user will look in the near future. Most existing 360 video streaming systems rely on current or past head orientations to predict head movements, but they suffer from low streaming efficiency because fast and sudden head movements are too unstable to predict by the history [6,10,11,12].

It is well known that understanding human visual attention to 360 videos is the key to improving head-movement prediction [13,14]. By leveraging the knowledge of *salient regions of interest* where users tend to view coupling with the user’s *head movement history*, the accuracy of the head-movement prediction for 360 videos can be improved. A 360 video streaming system employing this double-channel head movement prediction would be able to reduce its spatial fetching error, allowing users to enjoy downloaded content in high quality and improving the Quality of Experience (QoE). The key steps to achieve such a *saliency-based* 360 video streaming system include (a) a proper saliency detection model for 360 videos, (b) a unified approach integrating salient regions with head movement history to achieve accurate head-movement prediction, and (c) an efficient streaming system that only streams the salient content by exploiting the head movement prediction results.

Despite this consensus, the vision of improving 360 video streaming with saliency-enhanced head movement prediction has never been realized. All previous works are only able to achieve one or two steps of the aforementioned saliency-based streaming system. Some works have proposed saliency-based head-movement prediction models, but they did not design and evaluate the end-to-end streaming system in a real-world context [13,14,15]. Some streaming systems utilized a single-saliency channel for head-movement prediction without considering the impacts of head-movement history on streaming performance [16,17]. Others used improper video saliency features derived from 360 images [18] or regular 2D videos [14,19] rather than 360 videos that present unique salience patterns. While prior works provide important insights, they lack a holistic approach to identify the unique saliency of users in 360 video viewing, then using the saliency to construct an enhanced head-movement prediction model, and finally incorporating the model as an organic part of a streaming system. As a result, their streaming performance is negatively affected.

In this paper, we propose a streaming framework to unleash the potential of the salient content in 360 videos. The key is to capture the unique viewing attention of users on 360 videos inside VR HMDs. By supplementing important visual features to the head movement predictor, we aim to ultimately enhance the overall performance of the streaming system. We face several challenges to achieving this goal.

First, when training the 360 video saliency detector through deep neural networks, overfitting can happen easily if the training set lacks variety. While increasing the richness of the training data helps, user behavioral data in 360 videos are limited compared to regular 2D videos and are expensive to collect. Hence, we propose to use transfer learning, where the parameters from models previously trained on regular videos are transferred to our model. This allows our model to inherit knowledge about common visual patterns from much larger datasets [20] in domains similar to 360 videos. Our approach significantly reduces the number of required samples from several thousand [20] to several hundred.

Second, the predicted saliency may not always indicate the head movement of users. This is because a 360 video can have multiple salient regions evolving through time. It is not straightforward to identify the salient content to be viewed by the user hidden among false-positive noises. To cope with this challenge, we leverage the highly non-linear structure of the Long Short-Term Memory (LSTM) network to build the head-movement predictor by exploiting the temporal–spatial input of both saliency maps and head orientations.

Finally, the impacts of head-movement prediction on the performance of the streaming system have not been studied comprehensively. Prior 360 video streaming research either demonstrates the ultimate streaming performance without isolating other design factors such as new video encoding methods and updated adaptation algorithms [6,11,21,22], or focuses on evaluating their proposed head predictors without placing them in the context of the streaming systems [14,23]. To fill this gap, we focus on the system aspects directly affected by the head movement predictor. The core of our system is a scheduler that selects the video region to be streamed and displayed only based on the prediction results from the head movement predictor.

We validate the proposed system with extensive evaluations using viewing logs of HMD users and LTE bandwidth traces ranging from 8 Mbps to 44 Mbps. Results show that under the normal bandwidth condition at 22 Mbps, the proposed 360 video streaming system reduces the stall duration by 65% and the stall count by 46% while saving 31% more bandwidth compared with systems using head movement predictors that do not utilize salient content.

Our contributions can be summarized as follows:

- A deep convolutional neural network (DCNN) that detects the unique saliency of 360 videos in HMDs.
- An LSTM-based head-movement predictor leveraging both salient content and users’ head-orientation history.
- A system that enables saliency-based 360 video streaming and studies the effect of head-movement prediction on the streaming performance.

## 2. Related Work, Motivation, and Overview of the Proposed System

### 2.1. Saliency Detection

Saliency detection is an important topic in the study of human visual attention. Early works focused on constructing saliency models using hand-crafted features [24,25]. However, their accuracy is limited by the heuristic design and the domain knowledge of the researchers. With the advancement of deep learning, extensive efforts have been made towards building neural networks that can learn and represent salient patterns directly from images [26,27,28]. These models are designed for regular images and videos, which are expected to be viewed on smartphones and computer screens. Thus, they do not apply to 360 videos displayed in VR HMDs.

Saliency detection for 360 images/videos has attracted attention recently. Researchers applied a 2D-image-based saliency detector on different parts of a 360 image and linearly combined results to produce the final 360 saliency map [29,30]. Others adapted the architecture of traditional saliency models to 360 images [31]. Efforts were also made to reduce the distortion of mapping 360 images to the equirectangular format and improve saliency detection [32]. These works, however, focused on capturing visual attention when viewing static scenes. Subjects were allowed to go back and forth along the 360 image as many times as possible to “find” the salient objects. This is in sharp contrast to 360 video viewing, where users may easily miss certain objects during their head movement because the content dynamically evolves over time. Few works have been proposed to address saliency in 360 videos. In [18,33,34], the authors aimed at improving the saliency detection accuracy near the poles of the 360 sphere by reducing the impact of distortion at the top and bottom of the equirectangular format. Others [35,36] converted the input 360 video into a cubic map to reduce the negative impact of distortion at the poles of the equirectangular and presented an attention architecture to increase the saliency detection accuracy.

Unlike prior approaches that better handle distorted objects in 360 video saliency detection, we aim to enhance saliency detection by capturing unique visual attentive behaviors of humans in VR HMDs. Our preliminary results have discovered and addressed two types of unique head behaviors related to visual saliency on 360 videos [13]. First, the equator bias indicates the user’s tendency to equally pay attention to the content regions near the equator of an equirectangular 360 video. Second, the multi-object confusion shows that users cannot view all salient objects as in 2D videos because these salient objects may be separated into different 360 video viewports that users can only see one at a time. In this paper, we extend our previous modeling work to end-to-end system design. We integrate our model and design several other components to enable a saliency-based 360 video streaming system. We also validate the whole system’s design in real-world system evaluations.

### 2.2. Head Movement Prediction

Head movement prediction is an essential component for an efficient 360 video streaming system. Head trajectory history, a series of time-stamped quaternions representing the orientations of the users’ heads, plays a pivotal role in predicting future head movement. The processing and analyzing of head trajectory history in different ways have been used in previous head movement prediction models. Early models reused current head position [37] or conducted basic processing of the head movement history, such as linear regression [22,38], weighted linear regression [21,39,40], or other simple transformations [41]. Recently, efforts have been dedicated to developing more advanced algorithms. For example, LSTM-based [42,43,44] and CNN-based [45] networks receiving the past history of viewports were employed to predict future viewing angles. In [15], the prediction was based on the target user’s viewing history and the future viewing knowledge from other users. Unfortunately, these aforementioned single-modality models only explored head trajectory history for the prediction and their performance is unreliable under the fast and sudden head movement in HMDs.

To address this limitation, video content features have been used as another approach to enhancing head-movement prediction. Aladagli et al. predicted head movement based on a pre-trained saliency model by other researchers [23]. Fan et al. combined saliency and prior head orientations to further improve the prediction accuracy [14]. However, both schemes rely on traditional saliency models for regular 2D videos, which cannot capture the unique visual attention in 360 videos. In [46], the authors supplemented their predictor with small video patches around the most recent head position. Unfortunately, the small size of patches has a low coverage of content and would prevent the model from anticipating large head movements, which are the main source of head prediction errors. A saliency predictor trained on 360 images was used to assist head movement prediction [18]. However, it cannot capture the distinct human behavior when exploring 360 videos, which affects the performance in streaming salient video regions. In [47], object detection results were used as supplemental features for head movement prediction, but its streaming performance is limited by the number of object classes and the mismatch between saliency and objects.

Unlike these works, we employ both factors affecting head movement, i.e., the saliency crafted for dynamic 360 videos and the user’s head movement history, and then explore the interplay between them, to maximize the head movement prediction performance and enhance the 360 video streaming system.

### 2.3. 360 Video Streaming Systems

Unique challenges are presented by 360 video streaming. The occurrence of lagging and stalling leads to motion sickness [48] that breaks the immersiveness [49]. Therefore, high-performance streaming system is important to maintain users’ QoE. Various 360 video streaming strategies have been proposed. Two-tier streaming [22] used a base-tier buffer to stream the whole video frame at the lowest quality level. A spatial region encoded in a higher quality that covers the user’s current viewport was fetched to the enhancement-tier buffer. However, the data streamed to the two buffers compete with each other for available bandwidth, degrading the overall system performance.

In viewport-adaptive streaming [6], multiple versions of a equirectangular video [50,51] were prepared at the server. Each version had a small selected area encoded in higher quality. The version where the high-quality area closely matched the predicted viewport was selected for streaming. This approach has a high storage demand at the server since each version is always a full equirectangular video.

Tile-based streaming [12,52,53,54] is the most popular approach for 360 video delivery. A video chunk is spatially cut into tiles of equal sizes, and only relevant tiles are transported to the client. Tile-based approaches do not have redundant information in viewport adaptive streaming or two-tier streaming. There are different approaches to leverage tiles. For example, tiles can be ranked based on the distance to the center of predicted viewports [10] and then selected based on bandwidth conditions, buffer status, and video quality constraints [10]. Other works combined tile-based streaming with a scalable video encoding method [11,55,56], which split each tile into multiple quality layers. In addition, deep learning techniques such as deep reinforcement learning were used to adaptively allocate tile bitrate [42]. We focus on the tile-based approach, since it is more practical regarding server-side storage.

Despite the insights of previous works, they overlook the direct effects of the head movement predictor. The results reported before often showed the overall system performance, which is the result of many factors such as encoding schemes [6,11,22], tile planing [10], buffer management [22], and neural enhancement [42]. In other cases, only the accuracy of the head movement prediction algorithm is evaluated without putting it in the real-world streaming context [13,57]. In this paper, we instead build our streaming system centered on head-movement prediction to highlight the impact of the proposed saliency-driven head-movement predictor.

### 2.4. Overview of the Proposed System

**Figure 1:**
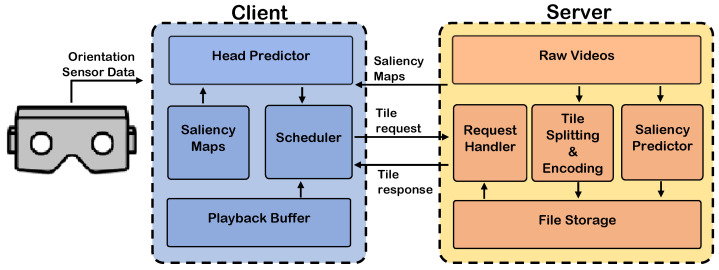
The proposed saliency-based 360 video streaming framework.

In this section, we discuss the overall design of the proposed saliency-based 360 video streaming system. As illustrated in Figure 1, the system includes a client and a server connected via wireless/4G/LTE networks. The server is a traditional stateless HTTP-based video streaming server. Inside the server, the saliency predictor is one of the core components. On the client side, the head movement predictor and the scheduler are the two primary components.

The general interaction among these components is as follows. Saliency maps of 360 videos are generated offline at the server by the saliency predictor. During the session initialization, the saliency maps are transferred over the network to the client before the 360 video data are streamed. At the client, the head predictor utilizes saliency maps and head movement history from the HMD to predict where the user will look in the near future. The scheduler receives the results from the head movement predictor and produces a spatial fetching decision to request the actual 360 video data. Upon receiving the client request, the server responds with the salient 360 video regions for the user to enjoy.

Our end-to-end design is a holistic framework including all essential components to realize the idea of saliency-based 360 video streaming. The design of the client highlights the interaction between the head movement predictor and the scheduler, allowing us to investigate the effect of head-movement prediction on system performance. In the following, we will introduce the details of the server and the client, especially the core components.

## 3. Video Streaming Server

We start by discussing the workflow, introducing how the saliency predictor and other components in the server interact. Then we go into the details of the saliency predictor, the core component of the server.

### 3.1. Workflow

The server has two main tasks, preparing video data and metadata offline and responding to tile requests with video data online. During the offline streaming preparation, the raw 360 video is first temporarily divided into chunks. Each chunk is then spatially cut into tiles of equal size. The tiles are encoded and stored as files in the permanent file storage. The spatial and temporal positions of each tile within the 360 video are recorded so that the server can locate them at a later time. Meanwhile, the server utilizes *PanoSalNet*, the saliency predictor to be introduced in Section 3.2, to identify the saliency maps of the raw video. The saliency maps are also saved in the file storage.

At the beginning of the streaming session, a Media Presentation Description (MPD) file that contains positioning information about every tile is sent to the client. The positioning information includes the chunk index number and the spatial coordinates of the tile in the chunk. The saliency maps are also sent to the client along with the MPD file. The saliency maps are rescaled to a smaller size to reduce their bandwidth consumption. During the streaming, the Request Handler module waits passively for tile requests from the client. The tile request specifies a list of tiles and the associated positioning information. The server locates the tile files and streams them back to the client. Similar to traditional video streaming servers, the Request Handler is stateless; i.e., it does not store any status associated with any client, making it easy to scale up to a large number of requests.

### 3.2. 360 Video Saliency Detection Model

As we discussed, the attentive behaviors of users in 360 video viewing under HMDs differ from those of 2D videos/images and 360 images. Since it is not possible to collect ground truth saliency maps for every video through subjective study, we need a model that can learn unique human attentive behaviors captured in limited public datasets and perform saliency detection on new videos not present in the training. To achieve this objective, we propose PanoSalNet, a DCNN-based panoramic saliency detection model for 360 videos. DCNN has been the primary method in image processing [26,27,28] due to its powerful capability in learning the representation of highly complicated visual patterns and delivering superior performance in various image analysis tasks. Below, we introduce its network architecture and model training in detail.

#### 3.2.1. Network Architecture

In general, a large and diverse dataset is needed for training to avoid SM overfitted DCNN model and to produce a better DCNN performance. Considering the human-centric nature of visual attention studies [20], it is even unlikely in the future, if not impossible, to collect millions of saliency maps as in image classification. To address this issue, we propose employING transfer learning to adapt an existing model to the target DCNN for 360 video saliency detection using the limited public data of 360 video saliency maps. Transfer learning is a popular technique to bring pre-trained deep learning models into other expert domains that significantly reduces the amount of required training data. This technique has been successfully applied to problems with very small domain datasets [58,59]. A similar strategy has also been adopted in the training of saliency detection for 360 images [31,60].

**Figure 2:**
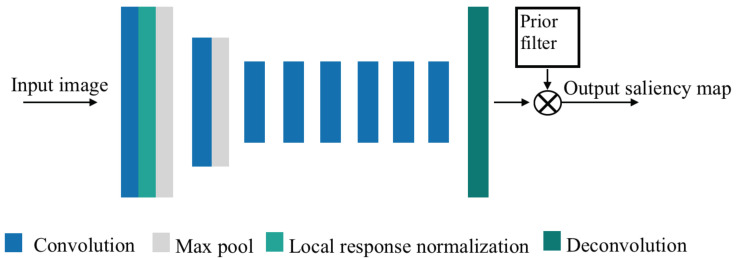
Deep neural network architecture of PanoSalNet.

The network architecture of PanoSalNet is illustrated in Figure 2. The proposed network architecture with nine (de)convolution layers is inspired by Deep Convnet, a state-of-the-art DCNN for saliency detection of regular images. The first three layers enjoy the same structure of VGGNet [61], which allows us to initialize the parameters of these layers by a popular deep learning network that has shown outstanding performance on image-classification tasks. The next five layers are initialized and trained from scratch on SALICON [20], a saliency map dataset for regular images. To apply transfer learning to such a network, the last few fully connected layers, which contain most of the network parameters, are usually removed and replaced by layers suitable for the requirement of the new application domain [27]. However, since this architecture from Deep Convnet has no fully connected layer, we conduct transfer learning over all layers after the initial parameters of these layers are obtained, as mentioned above. This way, we adapt this traditional model to the proposed PanoSalNet.

The predicted saliency is enhanced one more time as the final output by a prior filter [62], which lowers the saliency in areas based on a priori knowledge, such as four corners of an equirectangular frame.

#### 3.2.2. Model Training

To expedite the model learning, the frame resolution was downscaled to 512 × 288. Since predicting a saliency map is a regression problem, we use Euclidean distance to measure the difference between ground truth saliency maps and the predicted results. The loss function is defined as follows:

(1) $$L=\frac{1}{N}\sum_{i}L_{i}\left(f\left(X_{i},W\right),y_{i}\right)+\lambda R\left(W\right)$$

where the batch size *N* is set to 3, *W* is a model parameter to be learned, $L_{i}$ is the Euclidean distance between the output saliency and the ground truth saliency $y_{i}$, and R(W) is the regularization expression. PanoSalNet uses standard $l_{2}$ for regularization to control overfitting. The *f* function calculates the output saliency map based on the input image $X_{i}$. During the training, PanoSalNet was tested every 100 iterations, and the training was stopped at 800 iterations to prevent overfitting.

After model training, the proposed PanoSalNet is then able to detect the saliency map of an input 360 video.

## 4. Video Streaming Client

We now discuss the streaming workflow at the client end, i.e., the function of each component and the interaction between them. Then, we introduce the two core components of the client, the scheduler and the head predictor.

### 4.1. Workflow

The streaming client is empowered by the interactions among three components, the scheduler, the head predictor, and the playback buffer. During playback, the scheduler periodically, or at specific events, calls the head predictor to retrieve the predicted user head orientations in the near future. Upon receiving responses from the head predictor, the scheduler sends tile requests to the server. Tiles received from the server are put into the playback buffer before displaying to the user.

#### 4.1.1. Playback Buffer

The client decodes and stitches the received tiles before putting them into the playback buffer. Each buffer item corresponds to a video chunk. The items in the playback buffer are taken out at a steady rate and displayed in the user’s viewport. The length of the buffer is shorter than buffers in regular 2D video streaming systems due to the uncertain nature of the head prediction task [22]. If the buffer is empty, an exception will be sent to the scheduler.

#### 4.1.2. Head Predictor

When the head predictor receives a request from the scheduler, it gathers input from two data sources and returns back a predicted head orientation map. The two data sources include the gyroscope sensor and the saliency maps initially received from the server. The gyroscope provides head movement data points in real time, which are converted into input head orientation maps. Depending on the type of request from the scheduler, the head predictor could either return the head orientation map corresponding to the current head position or employ the LSTM architecture discussed in Section 4.3 to predict head orientation maps of the future positions. While future head positions are used in most cases, the scheduler requires the current head position when exceptions occur. Details about the exceptions are discussed below.

#### 4.1.3. Scheduler

The scheduler utilizes the result of the head predictor to request tiles such that the bandwidth is used in an efficient manner while ensuring the QoE. Its job includes forming a tile plan, sending tile requests, managing the buffer content, and handling exceptions. Exceptions occur when the buffer is empty or when the user’s head moves out of the planned tile area.

### 4.2. Streaming Scheduler

We proceed to introduce the proposed scheduler that highlights the impacts of head movement prediction on 360 video streaming.

#### 4.2.1. Tile Planning

The purpose of tile planning is to prepare a list of tiles to request. The set of tiles should cover the entire HMD screen while being small enough to not waste valuable bandwidth. To this end, our strategy is to select enough tiles to cover the predicted viewport and the additional margins in all four directions (up, down, left, right). As most prediction errors are the small misalignment between the predicted and the actual viewports, it is possible to choose an appropriate margin size to cover this mismatch without consuming excessive bandwidth.

Let T be the set of all tiles that form the equirectangular of a chunk from the original video and r be a tile from T. Let ρ be the tile planning function that receives a head orientation map x as input and returns a set of tiles. Tile planning is formally defined as follows,

(2) ρ(x)={r∈T|D(r,ξ(x))<e}

where ξ(x) is the tile that overlaps the most salient pixel in the head orientation map x. e is a controllable parameter deciding how many tiles the set would have. D(r,ξ(x)) returns the distance between the given tile r and the center tile ξ(x). The distance between r and ξ(x) is calculated as

(3) $$D\left(r,\xi \left(x\right)\right)=\max_{i,j}\left(\right|r_{i}-\xi {\left(x\right)}_{i}\left|,\right|r_{j}-\xi {\left(x\right)}_{j}\left|\right)$$

The *i* and *j* indices denote the row and column positions of a tile in the equirectangular. The underlying idea is that the scheduler first finds a center tile corresponding to the most salient region from the head orientation maps and then adds tiles staying the closest to the center tile to the list of selected tiles until the list reaches the desired size.

#### 4.2.2. Handling Unexpected Head Movement

In extreme cases, a wide and unexpected head movement could bring the viewport outside the prepared tiled area. Such a case has a detrimental effect on the user QoE as they will see the many blank tiles occupying a large portion of the viewport for a period of time. In this case, stalling the playback is a straightforward approach to prevent users from losing track of the video content. This is similar to the rebuffer in regular video streaming, where playback stalls when there is no content to display.

**Scheduling Algorithm.** The aforementioned behavior of the scheduler is summarized in Algorithm 1.
**Algorithm 1:** Tile scheduling algorithm
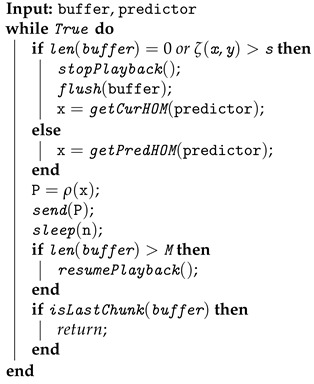


To make the decision of stalling the playback, we need to quantify the ratio of the blank screen area inside the viewport. To this end, we first convert the actual viewport into a tile presentation. Let the tiled version of the actual viewport, denoted by Λ, be the smallest set of tiles such that the viewport is covered. If the number of tiles for the equirectangular is large enough, the size of the tiled version will be close to the size of the actual viewport. Let the blank screen area be the set of blank tiles displayed inside the user viewport due to a head prediction error. Then, the Blank Ratio for unexpected head movement is calculated using the following formula:

(4) $$\zeta \left(x,y\right)=\frac{\left|\Lambda (x)\backslash \rho (y)\right|}{\Lambda (x)}$$

In the formula above, x and y are head orientation maps corresponding to actual and predicted head orientations of the user. ζ(x,y) is a function that receives two head orientation maps as the input and calculates the ratio of the number of tiles belonging to Λ(x) but not ρ(y) to the total number of tiles in Λ(x). We can quantify the stalling event due to the appearance of a blank screen by defining a threshold s; i.e., the stall happens when

(5) ζ(x,y)>s

To remedy the issue of unexpected head movement, we employ the flush and resend strategy. When the Blank Ratio is greater than a threshold s, the scheduler stops the playback, cancels the ongoing tile request, and plans a new set of tiles around the current viewport. Ongoing requests are canceled due to the fact that extreme head movements make ongoing transmitted tiles no longer useful as the majority of the tiles will not be shown to the user.

#### 4.2.3. Handling Buffer Starving

Users see a totally blank screen when the buffer is empty. This happens when bandwidth is insufficient, causing requested tiles not to arrive at the buffer on time. Due to the dynamics of the head movement, the user’s action during this period is not defined. Relying on ongoing requests from previous head predictions is not reliable. In this case, the scheduler should reset the tile request with the current head position. Note that we design this buffer management scheme to more effectively investigate the impacts of head movement prediction on the system performance in bandwidth-limited networks. By using a relatively simple scheme, we aim to isolate other system design factors and focus on head movement prediction.

### 4.3. LSTM-Based Head Movement Prediction

One key obstacle for head movement prediction in previous works is when the user takes a fast head movement, during which the prediction accuracy has been observed to drop significantly [21]. This type of fast head movement can occur when a new object is presented. Since a user typically moves her head to the most salient region of the video, a more accurate saliency detection can potentially address the fast movement prediction and improve the head movement prediction. Hence, we integrate the 360 video saliency maps generated from PanoSalNet with user head orientation history for head movement prediction. We exploit both factors and their interplay to maximize prediction accuracy.

#### 4.3.1. Model Architecture

In order to learn the pattern of head tracking logs of multiple users and to capture the interplay between temporal user behavior and multiple saliency maps from past video frames, a highly nonlinear learning model is required. We propose utilizing a Long Short-Term Memory (LSTM) model, since LSTM is proven to be able to handle a large amount of temporal data and outperform other algorithms [63].

**Figure 3:**
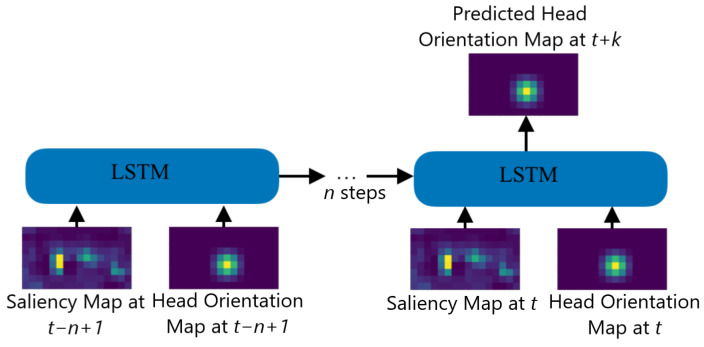
The proposed LSTM architecture.

In particular, the proposed LSTM model is a Recurrent Neural Network (RNN) that works on the temporal domain. Since stacking multiple layers of LSTM on top of each other can handle more complex data [63], we adopt an LSTM network with the model hyperparameters of two layers and 128 neurons per layer for the model training and validation. The network architecture of the head movement predictor is shown in Figure 3. The network receives input from a given number of previous time steps and provides the prediction of head orientation in the next *k* time steps (prediction window). The input features of the LSTM network include both the 360 video saliency map detected by the proposed PanoSalNet (indicating regions of interest) and the head orientation feature (recording head-movement history).

To better correlate head orientation with requested/viewed video tiles in 360 video streaming, we follow a similar method in [14] and represent the head orientation feature by *head orientation map*, a spatial data structure similar to a saliency map. Each pixel in the map corresponds to a video tile, with bright colors signifying proximity to the center of the viewport. Input head orientation map highlights the viewport within a frame that would be viewed under the current head orientation. We generate the head orientation map by first identifying the tile pointed to by the current head orientation vector and set its likelihood to be viewed as 1.0. Using this tile as the center, we then apply a Gaussian kernel to gradually select other tiles of the viewport with a lower likelihood to be viewed around the center tile.

#### 4.3.2. Model Training

The loss function for training is calculated based on the Euclidean distance between the predicted head orientation map and the ground truth head orientation map. Our model parameters are updated with Root Mean Square Propagation (RMSprop) method, a preferred method for RNN as suggested by [64].

## 5. Evaluation

In this section, we conduct experiments to investigate the performance of the streaming system. We start by describing our experiment setup. Then, we detail the evaluation results.

### 5.1. Experiment Setup

Here, we describe datasets used to train the saliency predictor and the LSTM-based head movement predictor. Then, we discuss the setup and evaluation of our trace-driven 360 video streaming system.

#### 5.1.1. Training Datasets

**360 Video Saliency Dataset.** To train the saliency predictor, we use an existing saliency dataset for 360 video viewing [65]. Each saliency map highlights salient regions derived from HMD viewing traces of at least 48 users. Investigation of the saliency dataset shows that fixation points tightly cluster around the regions of interest when they initially appear. Then, they move around the original clustering point when some users move their heads away. We have found that such scattered fixation points would prevent the model from learning meaningful patterns and would significantly degrade detection performance. Thus, we selected 400 pairs of video frames and saliency maps from the saliency dataset to conduct the transfer learning of PanoSalNet. We also guarantee that there are not too many similar frames for the same video scene, which prevents overfitting on a few video scenes with a large number of frames. Note that the number of data is sufficient and is consistent with previous models using transfer learning that have 40–1000 frames of domain data [58,59,60].

**Head Movement Dataset.** To train the head predictor, we use saliency maps output from the saliency predictor and head traces from five videos from [13]. For each video, we select one segment of 20–45 s. The video segment is selected such that there are one or more events in the video that introduce new salient regions (usually when a new video scene is shown) and lead to fast head movement of users. We extract the time-stamped saliency maps and head orientation maps from these videos, creating a total of 300,000 samples from 432 time series using the viewing logs of 48 users.

#### 5.1.2. System Settings

**Real-world Traces.** The system receives head-movement traces from real users watching 360 videos in a free viewing context. Traces of 10 users are randomly selected from the 48 users viewing eight videos in 2K resolution [66]. Since there is a small difference in perceptual quality between 2K and 4K video versions [67], 2K resolution is acceptable. We emulated the variation in network condition by five 4G/LTE bandwidth traces selected from [68]. We selected 4G/LTE because it is a popular network [69] and presents a challenge for 360 video streaming due to its limited and fluctuating bandwidth. The bandwidth traces were collected in a representative 4G/LTE network, where the latency of the network was 40 ms, the bandwidth was 10 MHz, and the frequency was 2.6 GHz. The downlink and uplink speeds were recorded at 57 Mbps and 12 Mbps, respectively. We derived the granular throughput per second from bandwidth logs. The bandwidth logs showed the available bandwidth when smartphone users were traveling by bus or car or on foot on different routes in urban areas. The average value of granular throughput was 30.3 Mbps. To configure different conditions of the network, we also scale the mean values of these bandwidth logs to three levels: 8.5 Mbps, 22.5 Mbps, and 44.5 Mbps.

**Other System Parameters.** We set the chunk length to one second such that the system would not commit to one viewport for an extended period during which the user may have already changed the viewing direction. To balance the encoding efficiency and bandwidth efficiency, the number of tiles per chunk has been configured in a range from a half-dozen [21] to a few hundred [14,52]. We take the middle ground and set the tile size in our system to 9×16 tiles. The buffer size is set to be 3 s. The playback starts/resumes when the buffer has at least M=1 second of streamed data. The trained saliency predictor performs prediction to produce saliency maps for all video frames. The saliency maps are rescaled, and their size is equal to the number of tiles, i.e., 9×16 pixels. We observe that this only increases the bandwidth consumption by 0.8% when streaming one-minute-long 2K videos taken from [66]. We denote the number of tiles to be selected by the scheduler after performing the tile planning task ρ(x) as the Expanded Tile Size. It includes tiles covering the viewport and its margin. To explore the performance, the Expanded Tile Size is varied from 5×5 tiles to 11×11 tiles. During streaming, if the blank area inside the user’s viewport is greater than 20%, the playback stops.

**Implementation.** The saliency detection was implemented using the Caffe library. The head movement prediction model is implemented with Keras. Both models are trained on Intel Devcloud, a cloud computing platform optimized for artificial intelligence. The utilized Devcloud machine has eight Intel Xeon Scalable 6128 processors, with a GPU equipped with Intel Xeon E-2176 P630 processors. For the streaming system, the system logic is implemented in Python 3.6. The program runs on an Ubuntu OS 18.04.6 LTS installed on a desktop machine with Intel i5-10300H 2.5 Ghz, 16 Gb RAM and a NVIDIA GTX 1660 Ti GPU. The desktop computer was manufactured by Dell Inc., Houston, TX, USA.

**Baselines and Metrics.** We use three baselines to validate our streaming system. Each baseline uses an existing head predictor while the streaming configurations are kept the same. The first baseline [38] (denoted by Lnregr) utilizes a linear regression model to process the input saliency maps and head orientation maps. The second baseline [16] only takes the current saliency maps as input. Here, we implement it using the saliency maps detected by our model and denote the baseline as PanoSalNet. The third baseline [70] (denoted by Full) does not need input from the head predictor since it always streams all tiles. The prediction window for all models is 1 s.

We ran the experiments with ten users watching eight 2K 360 videos. The video titles were randomly selected from two datasets collected by X. Corbillon et al. [71] and C. Wu et al. [66]. The videos in both datasets were gathered online by the authors of the datasets. These videos were previously recorded by various sources. The content covers a wide range of topics such as sports, news, and documentaries. The raw video frame rate varies from 30 fps to 60 fps, whereas the raw resolution ranges from 1920p to 3840p. For consistency, all videos in our experiments are rescaled to 1920p at 30 fps. We selected the first 60 s of each video, when users were engaging more with the content [72]. For each combination of one user and one video, we conducted 20 viewing sessions. Each session uses a different bandwidth trace and an Expanded Tile Size value. This results in 1600 experiments in total. For each experiment, the performance of the proposed system and baselines were measured against two aspects of user experience, i.e., the quality of video content and the video playback smoothness. The quality of video content is measured through Viewport Perceived Quality. The smoothness of video playback is affected by stalls caused by an empty playback buffer and missing content display due to users’ head movement, which would be evaluated by metrics such as Buffer Stall Count, Buffer Stall Duration, and Blank Ratio. Specifically, the Viewport Perceived Quality metric compares the quality difference between tiles from the actual viewport and the original content. Buffer Stalling Duration measures the number of seconds the playback has to stop to wait for the buffer to be filled up. Blank Ratio is the average percentage of blank areas inside the users’ viewport during the viewing session. These are popular metrics that have been used to investigate the QoE in previous works [10,11,14].

### 5.2. Evaluation Results

In this section, we present results evaluating system aspects, highlighting the importance of head movement prediction on enhancing the performance of a streaming system.

#### 5.2.1. Buffer Stalling Count

**Figure 4:**
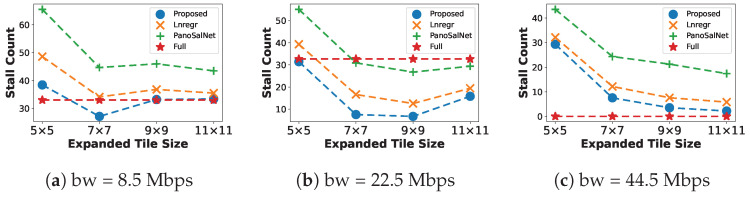
Buffer Stall Count under different bandwidth conditions.

Figure 4 shows the Stall Count during streaming under different bandwidth conditions. The proposed system shows its advantage as it consistently has a smaller Stall Count than most baselines at all bandwidth levels, thanks to its accurate head prediction model. Full tends to perform better than ours when bandwidth availability increases to 44.5 Mbps (Figure 4c). It achieves a near-zero stall count since it does not have any head prediction error. Even so, we observe that the performance of the proposed system approaches Full when the Expanded Tile Size is greater than 7×7, with only two stall events occurring at 11×11.

We also observe that low bandwidth (Figure 4a) generally causes the playback to stop due to buffer starvation. Moderate bandwidth (Figure 4b) only supports fetching spatial regions of average size (either 7×7 or 9×9). Smaller fetching size at 5×5 tiles is susceptible to the head prediction error, while enlarging the tile number to more than 9×9 tiles would easily overwhelm the available bandwidth. Both cases lead to higher Stall Count numbers. When the network bandwidth is sufficient (Figure 4c), the performance of models is only affected by head prediction errors.

#### 5.2.2. Buffer Stalling Duration

**Figure 5:**
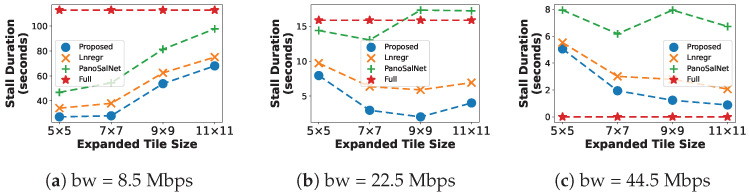
Buffer Stall Duration under different bandwidth conditions.

Figure 5 illustrates the Buffer Stalling Duration under different Expanded Tile Sizes. These results prove that our proposed head movement predictor is beneficial to the system as it incurs fewer stalls. In all cases, the proposed system has a much lower Stall Duration than other baselines in all Expanded Tile Size values. At moderate bandwidth, our system shows dramatic improvement, with the Stall Duration down to around two seconds at 9×9 (Figure 5b), which approaches the lower-bound performance of Full at high bandwidth (Figure 5c).

At low bandwidth (Figure 5a), the Stall Duration increases as the Expanded Tile Size grows. This is because the number of tiles quadruples as the Expanded Tile Size goes from 5×5 to 11×11, which significantly increases the time to download all requested tiles before the playback can resume. At higher bandwidth, larger Expanded Tile Size reduces stalls caused by head prediction errors. Specifically, Expanded Tile Size set at 7×7 or 9×9 is suitable for moderate bandwidth conditions (Figure 5b), while larger Expanded Tile Sizes should be used if the bandwidth is high (Figure 5c).

#### 5.2.3. Blank Ratio

**Figure 6:**
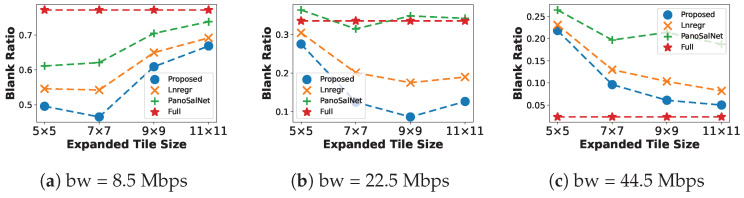
Average Blank Ratio for different head prediction models under different bandwidth conditions.

Figure 6 illustrates the benefit of using our proposed model. The accuracy of head movement prediction helps the system have a lower blank ratio in all Expanded Tile Size configurations at all bandwidth levels. Especially at a moderate bandwidth level (Figure 6b), our model outperforms the second-best baseline, the Lnregr, by a large margin of 10% when Expanded Tile Size equals 9×9. Increasing the bandwidth availability further improves our performance. In Figure 6c, as the buffer starvation is no longer a problem at 44.5 Mpbs, our model can utilize a larger Expanded Tile Size to approach the perfect performance of the Full baseline.

#### 5.2.4. Bandwidth Saved

**Figure 7:**
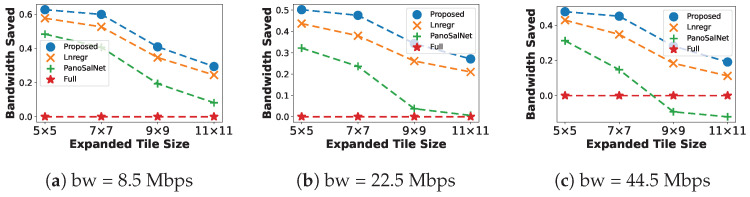
Percentage of Bandwidth Saved for different head prediction models under different bandwidth conditions.

The percentage of Bandwidth Saved (Figure 7) indicates how much bandwidth a system saves compared to downloading the whole equirectangular. Hence, the Full algorithm does not save any bandwidth and therefore serves as a lower bound. Figure 7 illustrates the advantage of using our proposed model regarding the bandwidth efficiency. Our system can save more than 60% bandwidth at 7×7 in low bandwidth (Figure 7a) and up to 37% at 9×9 in moderate bandwidth (Figure 7b). We conclude that the proposed head predictor helps save more bandwidth than other baselines while ensuring that the system maintains an appropriate performance level.

We additionally find that enlarging Expanded Tile Size reduces Bandwidth Saved since the number of request tiles to be sent over the network significantly increases. This explains the downward trends for all models at every bandwidth level. The accuracy of the head predictor also affects the percentage of Bandwidth Saved. Inaccurate models force the system to consume more bandwidth since it needs to resend tile requests over the network each time the head predictor makes an error.

#### 5.2.5. Viewport Perceived Quality

This section evaluates the quality of content perceived by users. Traditionally, the whole decoded content of the regular videos/images is compared with original videos using metrics such as PSNR and SSIM. However, this approach is not suitable for 360 videos since only the content displayed inside the viewport matters to users. Similar to works by [52,73], we derive Viewport Perceived Quality by using the SSIM score to measure the aforementioned difference between tiles from the actual viewport and the original content. To evaluate our proposed model under the Viewport Perceived Quality metric, we fix the Expanded Tile Size at 9×9 where the proposed LSTM has a balanced performance regarding Stall Count, Stall Duration, and Blank Ratio metrics.

**Figure 8:**
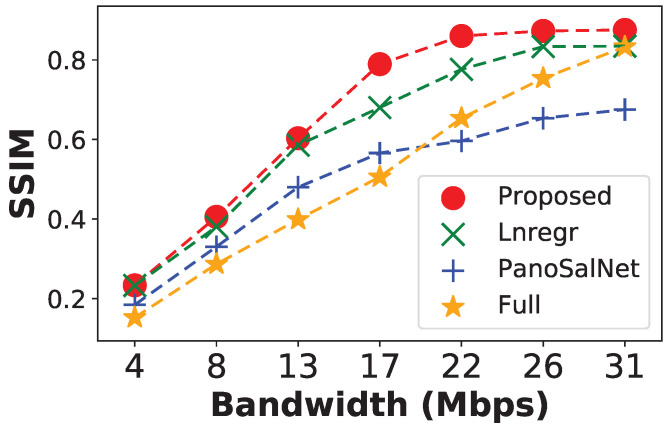
Viewport Perceived Quality under different bandwidth conditions.

Figure 8 shows the results under varying bandwidth conditions. When the bandwidth level is very low (less than 13 Mbps), all systems have low SSIM scores due to constant rebuffering, which causes users to experience blank screens regularly. From moderate bandwidth levels at 17 Mbps, the rebuffering effect is reduced. Thus, the viewport perceived quality for different systems becomes more dependent on the quality of their respective head predictors. The proposed head movement predictor helps the system attain significantly higher viewport perceived quality than other baselines. Moreover, our system reaches peak performance at 22 Mbps, while the nearest competitor, the Linregr, needs 26 Mpbs to reach its peak and its peak is at a lower score.

**Figure 9:**
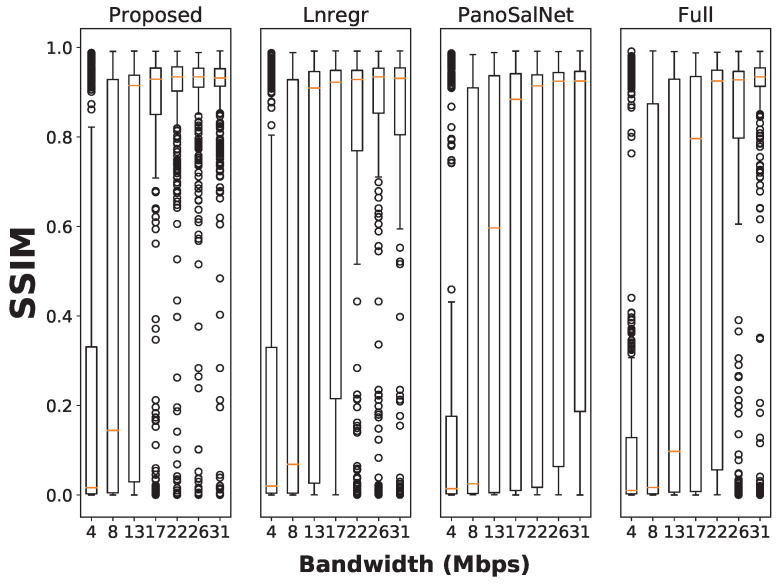
Box plot for users’ Viewport Perceived Quality under different bandwidth conditions. Each bar displays essential statistical information about the dataset, including the median, first quartile, and third quartile values. Outliers are identified by circles positioned above or below each bar.

We further investigate the distribution of viewport perceived quality scores for individual models at each bandwidth level and report the results in Figure 9. When the bandwidth level is at 17 Mbps or higher, the scores for our system are tightly clutched together at 0.9. On the other hand, the SSIM score distributions for linear regression are more spread out, with many outliers in the 0.0–0.2 range due to the less accurate head prediction. The score distributions of the Full baseline are even more spread out, especially when the bandwidth levels are low (less than 22 Mbps). The median score of our system is close to Full at 31 Mbps bandwidth. Note that the Full model tends to achieve perfect performance in higher bandwidth conditions where it is free from both rebuffering and head prediction error.

Combining both results from Figure 8 and Figure 9, we confirm that the proposed system significantly and consistently outperforms other baselines in Viewport Perceived Quality. The result proves that our head predictor helps the streaming system to deliver video in higher quality while only requiring a moderate amount of network bandwidth.

## 6. Limitations and Discussion

**Playback Buffer Size.** One of the key challenges in designing a 360-degree streaming system is the length of the playback buffer. It is not possible for 360-degree streaming systems to have more than a few seconds of buffer due to the high uncertainty of users’ viewing behavior. This uncertainty comes from the fact that users are the entities who ultimately decide where and what to watch, despite their tendency to focus on salient content. At any time, users may move their heads away from the prepared region, rendering all the downloaded data to become wasted. To overcome the issue and improve our system, the Expanded Tile Size could be dynamically increased/decreased according to the network condition. Ideally, the buffer content in the far future should cover more areas on the equirectanguler to accommodate any unexpected head movement.

**Bitrate Adaptation.** Bitrate adaptation is a popular technique that switches between different bitrate levels to adapt to the change in bandwidth conditions. With bitrate adaptation, the number of data can be varied depending on the bandwidth availability. This comes with a cost, however, since users may see time-varying video qualities between tiles. Currently, our system uses only one bitrate level since we focus on investigating the impact of the head predictor on the system performance. In the future, incorporating bitrate selection into our system will allow the system to stream 360 videos even in low-bandwidth environments.

**Tile Planning.** The tile planning module selects tiles until they cover the whole projected viewport. The projected viewport is the projection of the user viewport from the spherical surface into the equirectangular frame, which is fixed in our case. In reality, the projected viewport grows when users look up or down near the pole region. Since our tile planning function relies on the fixed projected viewport, the system underestimates the number of requested tiles covering the projected viewport when users look up or down. However, our assumption still holds in the majority of cases since users spend most of the time looking at areas near the equator [66,71,74], where distortion is minimized. In the future, we will address the distortion of the projected viewport to reduce errors in tile planning and improve the QoE when users focus on the pole regions.

**Head Movement Prediction.** Recently, self-attentive models such as the Transformer have become a favorable choice for temporal domain prediction tasks such as language interpretation. The main advantage of attentive models lies in their ability to select important but less frequent patterns while disregarding more recurring patterns. This may be beneficial for head movement prediction tasks since head movements are spontaneous, which correlate with few data points in the past movement trajectory. However, Transformer has higher computational requirements and needs a large number of training data. The applicability of the Transformer is still unclear, and investigating its potential for the head movement prediction task is left for future study.

## 7. Conclusions

This paper demonstrates the feasibility of utilizing salient content uniquely present in 360 videos to ensure accurate head movement prediction and efficient 360 video streaming. Our system leverages unique salient features, capturing humans’ exploratory behavior to assist the prediction of the head movement of users using HMDs. A streaming system seamlessly integrates the head movement prediction into its frameworks to improve overall performance. We conduct trace-driven experiments to confirm the advantages of the proposed approach. Through evaluations, the system is shown to excel for other baselines in multiple respects, such as reducing stalling duration by up to 65% and stall count by 46%. At the same time, the bandwidth saved is 31% more, which helps the systems to be more robust against different bandwidth conditions. Our work is an important step toward bridging the performance gap of head movement prediction and improving the QoE in today’s 360 video streaming systems.

## Data Availability

Not applicable.
